# Supplementary material for: Virological and Genetic Characterization of the Unusual Avian Influenza H14Nx Viruses in the Northern Asia
Source: Viruses. 2023 Mar 11;15(3):734. doi: 10.3390/v15030734 (PMC10058565; doi:10.3390/v15030734)
Supplement: Supplementary file 1 [file viruses-15-00734-s001.zip › viruses-2102710-supplementary.pdf]

Table S1. Description of isolated strains of AIV of H14 subtype

Strain name	Collection date	Location	Host species (lat)	Subtype	Accession num.
A/Common_Teal/Chany_Lake/29/2019	2019-09-07	Chany lake, Novosibirsk region, Russia	Anas crecca	H14N3	400267
A/garganey/Chany_Lake/210/2014	2014-08-24	Chany lake, Novosibirsk region, Russia	Anas querquedula	H14N9	14854178
A/garganey/Chany_Lake/211/2014	2014-08-24	Chany lake, Novosibirsk region, Russia	Anas querquedula	H14N9	14853905

Table S2. Identity of isolated strains.

Strain name	Segment	Related strain	Identity, %
A/Common_Teal/Chany_Lake/29/2019 (H14N3)	PB2	A/mallard/Omsk Region/63/2019 (A/H3N8)	99.57
	PB1	A/chicken/Poland/004/2020 (A/H5N8)	99.27
	PA	A/mallard/Toguchin/13/2017 (A/H4N6)	99
	HA	A/sandpiper/Tomsk/112/2019 (A/H14N7)	98.65
	NA	A/mallard/Chany Lake/18/2018 (A/H1N3)	99.51
	NP	A/mallard/Omsk Region/63/2019 (A/H3N8	99.67
	MP	A/mallard/Chany Lake/18/2018 (A/H1N3)	99.9
	NS	A/White-fronted goose/South Korea/KNU2019-39/2019 (A/H7N7)	99.77
A/garganey/Chany_Lake/210/2014 (H14N9)	PB2	A/tufted duck/Georgia/1/2012 (A/H2N3)	99.19
	PB1	A/shoveler/Chany/82K/2014 (A/H3N8)	98.63
	PA	A/teal/Egypt/MB-D-125OP/2015 (A/H7N3)	98.61
	HA	A/goose/Karachi/NARC-13N-969/2014 (A/H14N3)	98.24
	NA	A/Anas platyrhynchos/Belgium/195_7/2018 (A/H11N9)	98.88
	NP	A/environment/Kamchatka/18/2016 (A/H5N5)	99.62
	MP	A/mallard/Chany/355/2016 (A/H1N1)	99.51
	NS	A/teal/Egypt/MB-D-621C/2016 (A/H7N9)	99.33
A/garganey/Chany_Lake/211/2014 (H14N9)	PB2	A/mallard/Chany/260U/2014 (A/H5N3)	98.33
	PB1	A/shoveler/Chany/82K/2014 (A/H3N8)	98.58
	PA	A/teal/Egypt/MB-D-125OP/2015 (A/H7N3)	98.61
	HA	A/goose/Karachi/NARC-13N-969/2014 (A/H14N3)	98.24
	NA	A/Anas platyrhynchos/Belgium/195_7/2018 (A/H11N9)	98.88
	NP	A/environment/Kamchatka/18/2016 (A/H5N5)	99.55
	MP	A/mallard/Chany/355/2016 (A/H1N1)	99.51

	NS	A/teal/Egypt/MB-D-621C/2016 (A/H7N9)	99.21

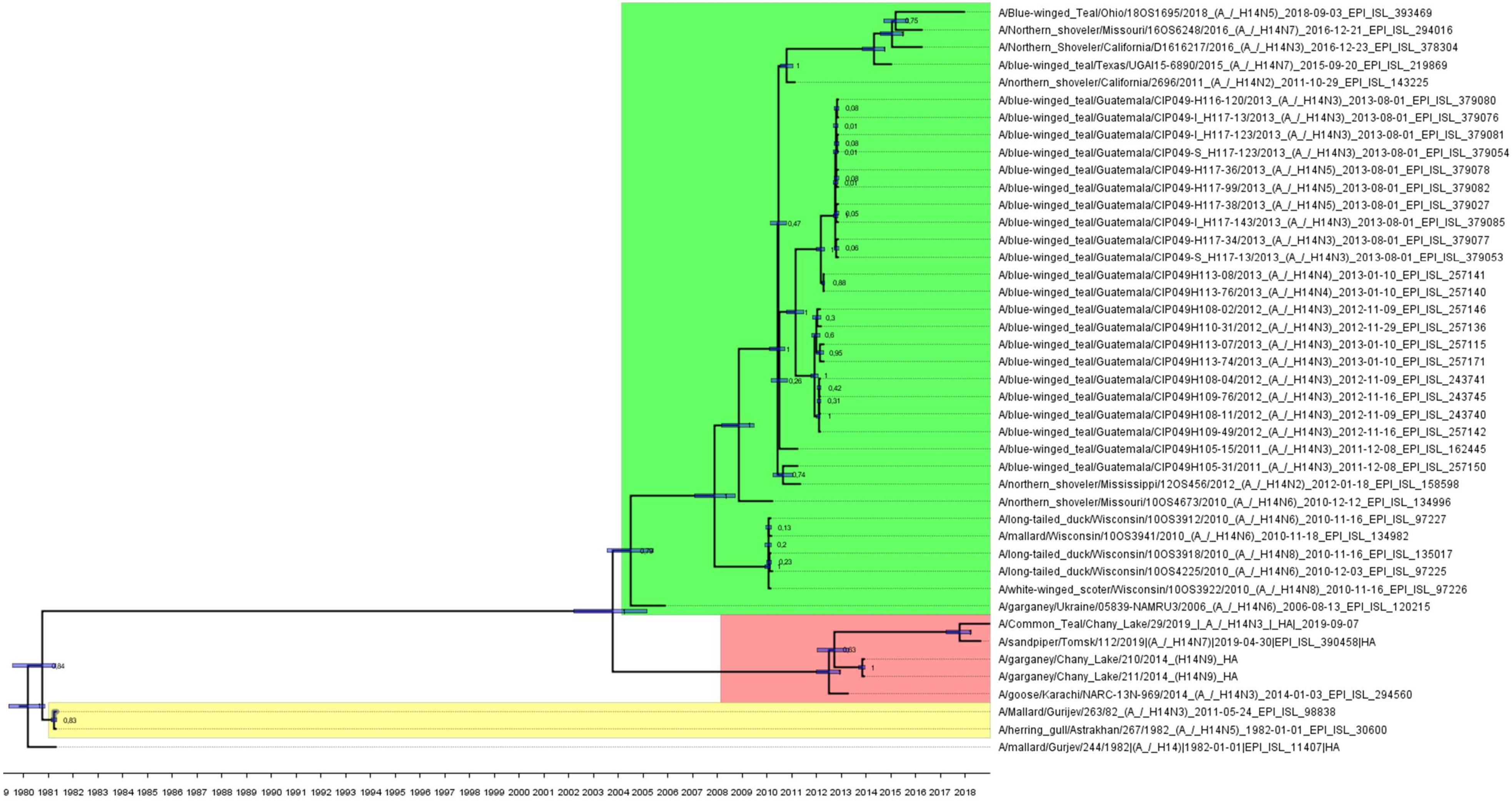

Figure S1. Time-calibrated phylogeny of nucleotide sequences of HA segments. H14.2.1 clade highlighted with red, H.14.2.2 clade highlighted with green. Node labels represent posterior probability, node bars represent 95% HPD interval.

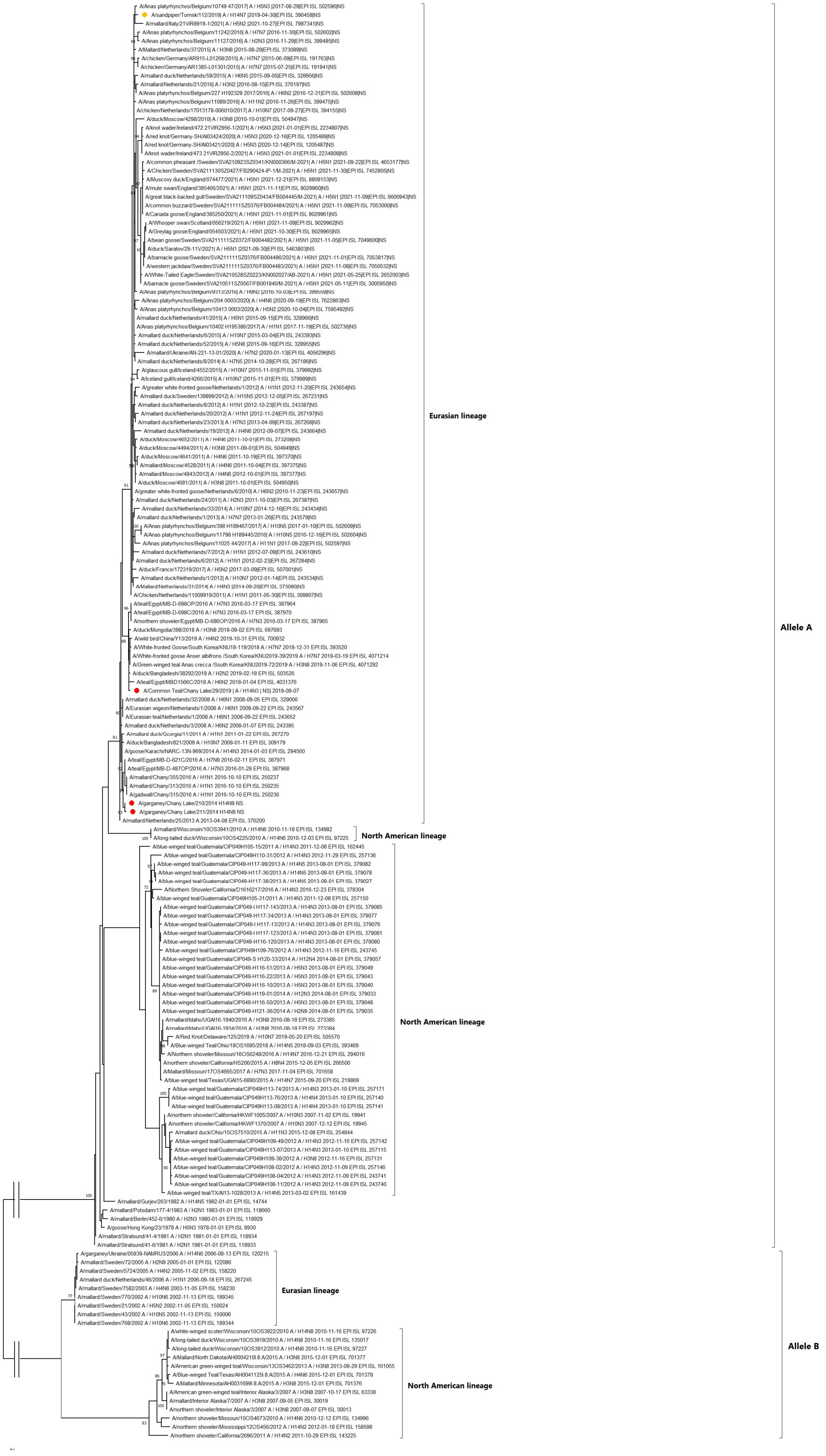

Figure S2. Maximum likelihood phylogenetic tree of NS segment nucleotide sequences (●—sequences from this study; ●—sequence of H14 influenza virus isolated in related region from public database).
